# Improving anti-melanoma effect of curcumin by biodegradable nanoparticles

**DOI:** 10.18632/oncotarget.20585

**Published:** 2017-08-24

**Authors:** Bilan Wang, Xiaoxiao Liu, Yan Teng, Ting Yu, Junli Chen, Yuzhu Hu, Na Liu, Lingli Zhang, Yangmei Shen

**Affiliations:** ^1^ Department of Pharmacy, West China Second university Hospital, Sichuan University, Chengdu, PR China; ^2^ Department of Pathology, West China Second university Hospital, Sichuan University, Chengdu, PR China; ^3^ Key Laboratory of Birth Defects and Related Diseases of Women and Children (Sichuan University), Ministry of Education, West China Second university Hospital, Sichuan University, Chengdu, PR China; ^4^ Evidence-Based Pharmacy Center, West China Second University Hospital, Sichuan University, Chengdu, PR China; ^5^ Department of Radiation Oncolo, Affiliated Hospital of Xuzhou Medical University, Xuzhou, Jiangsu, China; ^6^ Cancer Center, West China Hospital, West China Medical School, Sichuan University and Collaborative Innovation Center, Chengdu, PR China; ^7^ Research Unit of Infection and Immunity, Department of Pathophysiology, West China School of Basic Medical Sciences & Forensic Medicine, Sichuan University, Chengdu, Sichuan, China; ^8^ Puyang Medical College, Puyang, Henan, China

**Keywords:** melanoma, curcumin, micelles, cell apoptosis

## Abstract

Melanoma is known as the most common malignant cutaneous cancer. Curcumin, a natural component, has been shown to have various activities such as anti-oxidant, anti-septic, anti-inflammatory, anti-biotic, anti-amyloid and anti-thrombosis. However, there is a greatest obstacle in the administration of curcumin due to its hydrophobicity and low oral bioavailability. In this study, we formulated curcumin-loaded MPEG-PLA (Curcumin/MPEG-PLA) micelles aiming to improve its solubility in aqueous solution and investigated anti-tumor effect on melanoma *in vitro* and *in vivo*. The spherical curcumin/MPEG-PLA micelles were completely dispersed in normal saline and could release curcumin in a sustained manner *in vitro*. In addition, we demonstrated that curcumin/MPEG-PLA micelles had stronger cytotoxicity and induced a higher percentage of apoptosis in B16 and A375 cancer cells than free curcumin *in vitro*. The immunohistochemical study revealed that curcumin/MPEG-PLA micelles induced more melanoma cell apoptosis than free curcumin and inhibited neovascularization in tumor tissues. In conclusion, the curcumin/MPEG-PLA micelles have potential clinical application for melanoma.

## INTRODUCTION

Melanoma is a well known malignant cutaneous cancer. In the United States, melanoma is the fifth most common cancer among men and the seventh among women [1, 2]. The 5-year overall survival rate has reached 93%[1]; however, the overall prognosis of all the patients with distant metastatic melanoma still remains poor with median survival about 11 months [3].

Melanoma is highly resistant to conventional chemotherapy and radiotherapy. Thereby, novel immune strategies like cytokines, monoclonal antibodies, cancer vaccines, and adoptive T cell transfer are studied to improve prognosis of melanoma [4]. Among them, immune checkpoint blocker has become the remarkable highlight during recent years. Famous products, such as CTLA-4 blocker (ipilimumab) and PD-1 blocker (pembrolizumab, nivolumab), which have been proven to prolong survival time and approved by the US Food and Drug Administration (FDA) for clinical application [5–9]. However, the usage of these novel antibodies remains limited due to potential risk of immune related adverse effects (IRAE), involving dermatological, gastroenterological, endocrine, hepatic, pulmonary, ocular, renal, neurological and pancreatic systems [7, 10–14]. Thus, chemotherapy still plays an important role in the treatment of melanoma, and requires more strategies to decrease the related side effects and increase therapeutic efficacy.

Curcumin is a natural component extracted from traditional Asian spice turmeric with various potential activities such as anti-oxidant, anti-septic, anti-inflammatory, anti-biotic, anti-amyloid and anti-thrombosis [15–17]. Recently, researches have proposed its promising anticancer role in colon cancer, breast cancer, liver cancer, head and neck cancer, ovarian cancer, prostate cancer, non-small cell lung cancer, and pancreatic cancer [18–27]. Despite of being inexpensive and nontoxic, the unfavorable bioavailability which caused by poor absorption, quick metabolism and system elimination restricts the application of curcumin as an anti-cancer agent [17, 28, 29].

Nanotechnology has attracted much attention in drug delivery as nanocarriers can improve the water solubility of hydrophobic drug [28, 30, 31]. Poly (ethylene glycol) (PEG) is a non-toxic hydrophilic polymer widely used in drug carrier to solubilize drug [32, 33]. Meanwhile, poly (lactide) (PLA) is a popular biomedical material with good biocompatibility and biodegradable [34]. Nanoparticles assembled from the block copolymers composed of PEG and PLA have been extensively investigated for decades [35, 36]. Basically, a core-shell structure polymeric micelle is self-assembled with a hydrophobic PLA core and a hydrophilic PEG shell in aqueous medium through hydrophobic or ion pair interactions [37, 38]. Moreover, radical polymerization is applied to conjugate the double bonds at the end of the PLA blocks together establish a monomethyl PEG-PLA (MPEG-PLA) micelles [39]. The hydrophobic drug is encapsulated within hydrophobic PLA core of the micelles, covered by the water excluded PEG shell to obtain water solubility and slow release thereby prominently improve its bioavailability [40].

Therefore, it is interesting to combine nanotechnology and curcumin to create a desirable nano-vehicle for improving the anti-cancer effect and decrease side effects. In this article, we have successfully established an amphiphilic MPEG/PLA polymeric micelle encapsulating curcumin which showed sustained and controlled drug delivery *in vivo* and *in vitro*. Besides, we have further explored the anti-cancer efficiency and toxicity of this curcumin loaded MPEG/PLA micelles.

## RESULTS

### Preparation and characterization of curcumin/MPEG-PLA micelles

**Figure 1:**
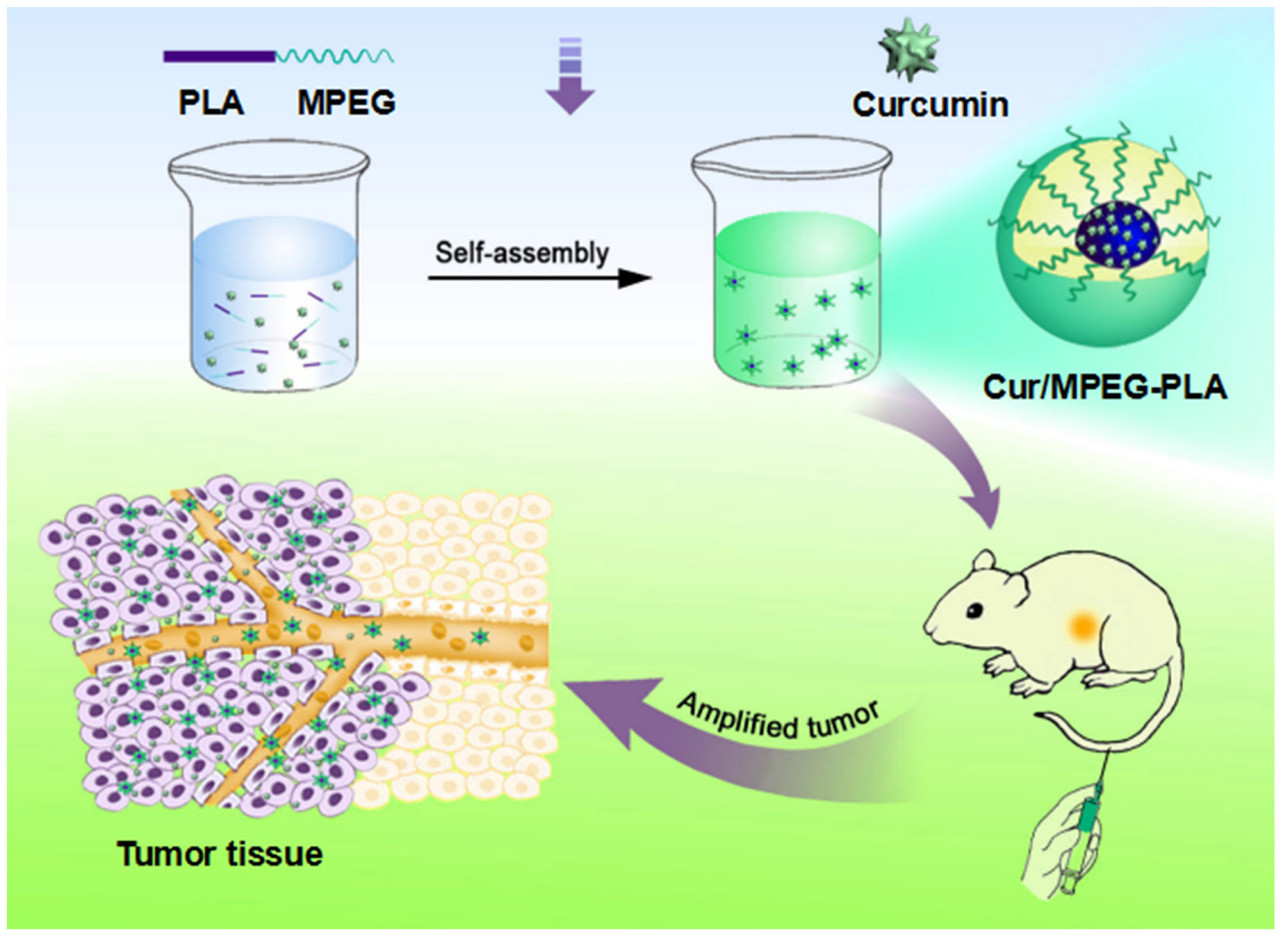
The picture of Curcumin/MPEG-PLA micelles preparation and tumor-burdened mice treatment Firstly, the Curcumin/MPEG-PLA micelles were prepared by self-assembly method. Then the mice with tumor was treated by curcumin nano-micelles. And the growth of tumor could be inhibited.

Curcumin/MPEG-PLA micelles were synthesized by a single-step precipitation method. Curcumin/MPEG-PLA micelles contains a ball-shaped hydrophilic PEG shell and a hydrophobic PLA core where curcumin was encapsulated into the core part of micelles and surrounded with PEG to improve its water solubility (Figure 1).

**Figure 2:**
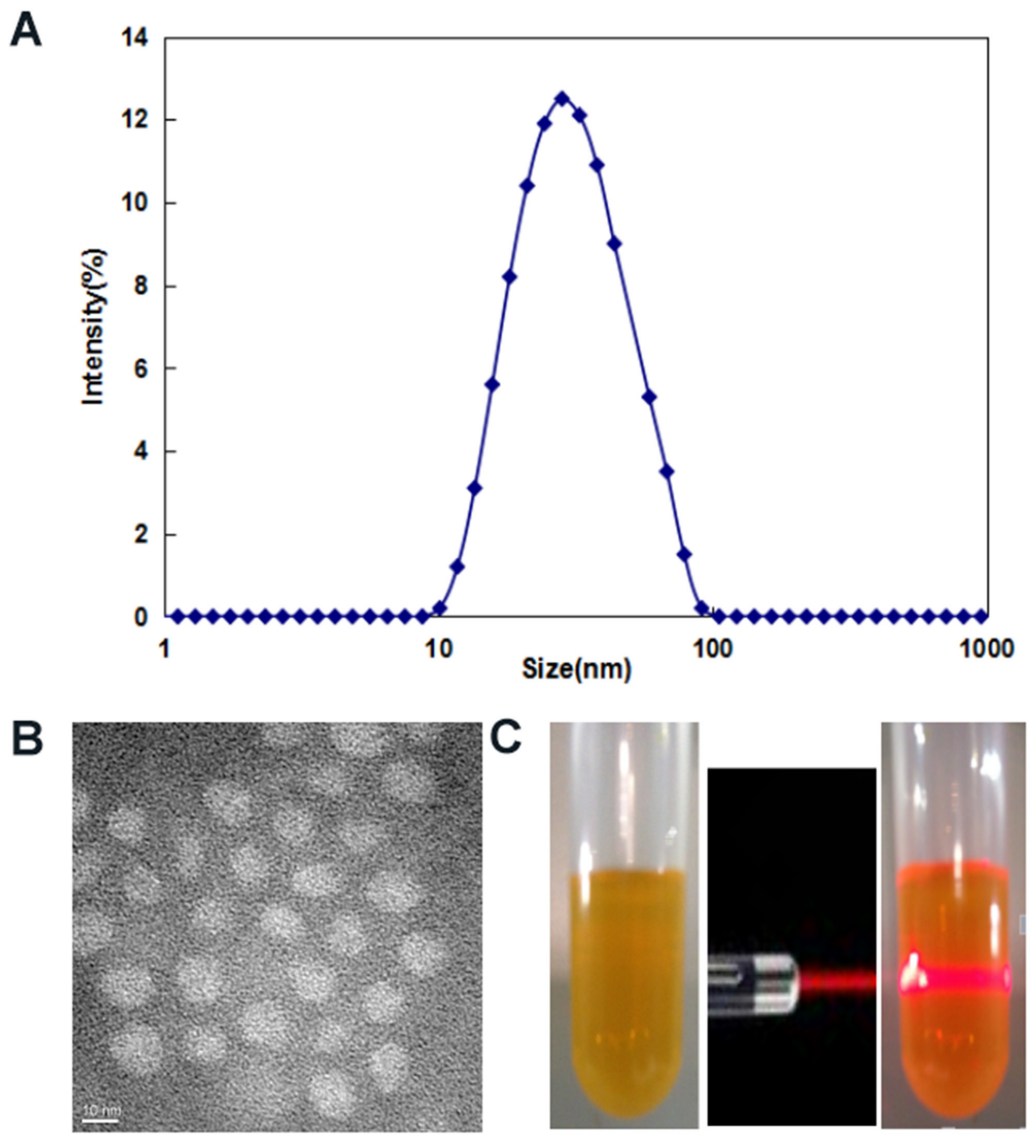
Characterization of Curcumin/MPEG-PLA micelles **(A)** Size distribution spectrum of Curcumin/MPEG-PLA micelles. **(B)** TEM image of Curcumin/MPEG-PLA micelles. **(C)** Photos of Curcumin/MPEG-PLA in normal saline solution and the Tyndall effect of Curcumin/MPEG-PLA micelles solution.

The drug loading (DL) and encapsulation efficiency (EE) of Curcumin/MPEG-PLA micelles were 10% and 98.8%, respectively. As shown in Figure 2A, the particle size of Curcumin/MPEG-PLA micelles measured by DLS was 34.5nm (PDI=0.13). Moreover, the size was confirmed by TEM (Figure 2B), which was consistent with the DLS results. Curcumin/MPEG-PLA micelles measured by DLS were in aqueous phase, therefore, the structure of these amphiphilic particles is usually loose in solution, and the DLS size is always larger than the TEM size. The surface of Curcumin/MPEG-PLA micelles was negatively charged, with the zeta potential of -2.3 mV. One of the major purposes of the encapsulation of curcumin in MPEG-PLA micelles is to make curcumin completely dispersible in aqueous media. As can be seen from Figure 2C, the solution of Curcumin/MPEG-PLA micelles was uniform and had a clear Tyndall effect, indicating the existence of abundant nanoparticles that were completely dispersed in aqueous media.

**Figure 3:**
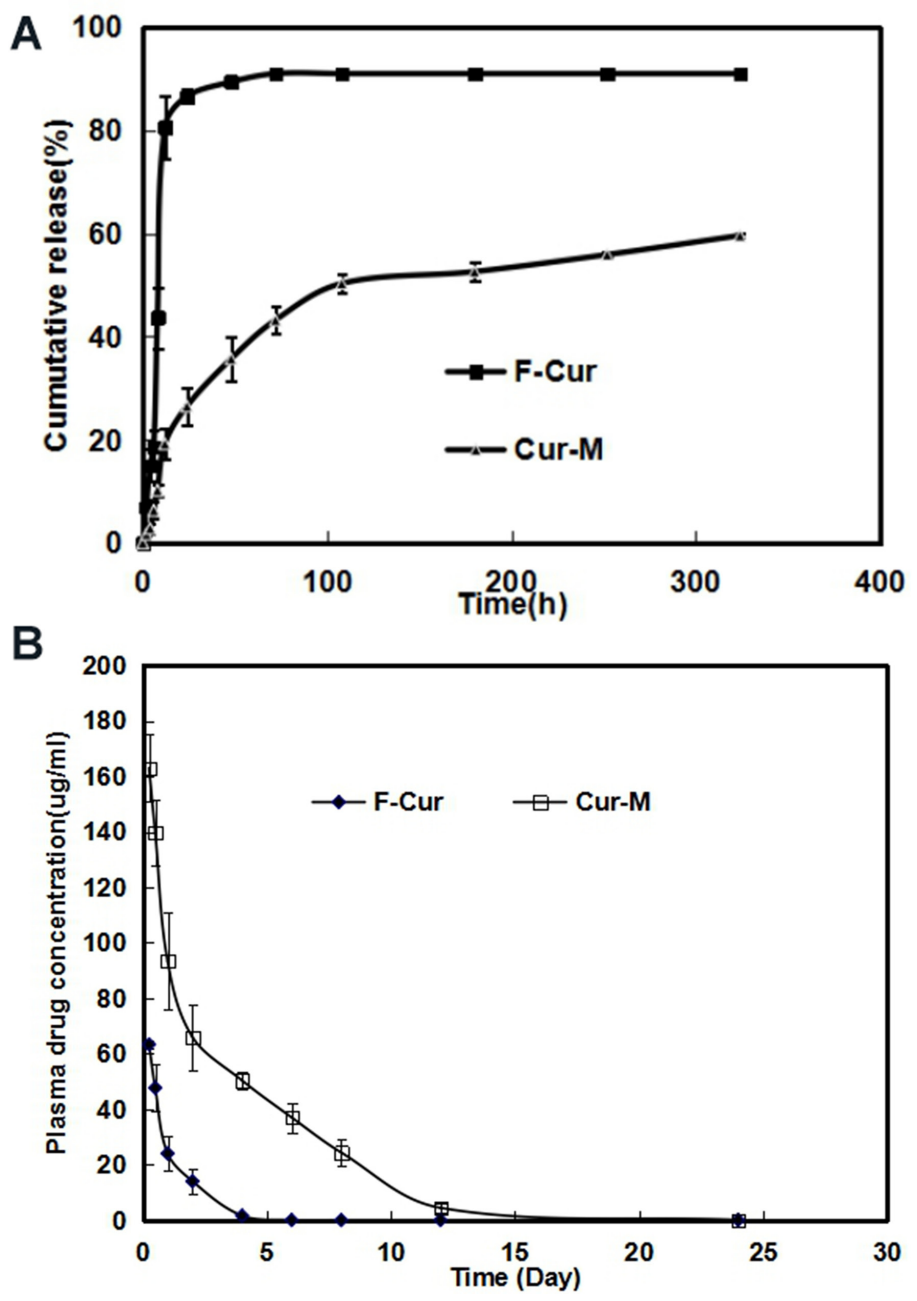
Free Curcumin and Curcumin/MPEG-PLA micelles release *in vitro* and pharmacokinetics assay *in vivo* **(A)** The curcumin release *in vitro*. **(B)** Pharmacokinetics assay of curcumin *in vivo*.

The *in vitro* release profile of free Curcumin and Curcumin from Curcumin/MPEG-PLA micelles was examined in PBS (PH7.4) at 37 °C. As shown in Figure 3A, free curcumin exhibited a rapid release and the accumulated release reached a peak of 80.6%±6.1% in 12 h. In comparison, only about 20% Curcumin was released from nano-micelles in the first 12 h and another 40% of drug was released sustainedly over 300h. The cumulative curve indicated that the drug-release process of Curcumin/MPEG-PLA micelles was stable and sustained *in vitro*.

Concentration–time profiles for free curcumin and Curcumin/MPEG-PLA micelles after intravenous injection were shown in Figure 3B. The T_max_, T_1/2_, C_max_ and AUC of free curcumin were 15 min, 0.89 h, 63.3 μg/ml and 65.9 mg L^-1^ h^-1^ respectively, while the T_max_, T_1/2_, C_max_ and AUC of Curcumin/MPEG-PLA micelles were 15 min, 1.65 h, 163.3 μg/ml and 498.5 mg L^-1^ h^-1^ respectively, suggesting that MPEG-PLA micelles could improve the bioavailability and pharmacokinetics of curcumin *in vivo*.

### Anti-melanoma effect *in vitro*

#### Curcumin suppressed cell viability

**Figure 4:**
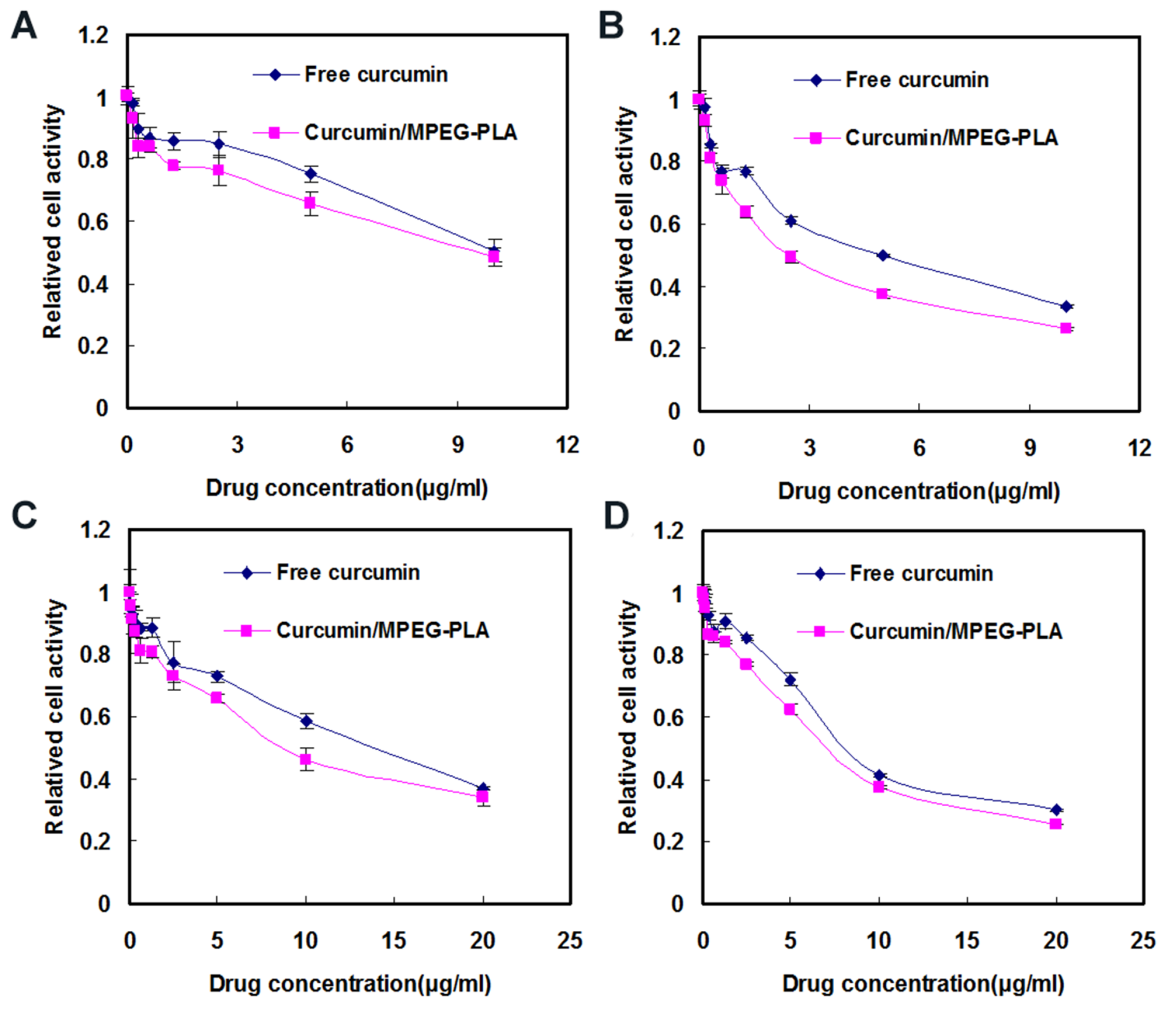
Cytotoxicity studies of free curcumin and Curcumin/MPEG-PLA micelles on B16 cells for 24h **(A)** and 48h **(B)**. Cytotoxicity evaluation of free Curcumin and Curcumin/MPEG-PLA micelles on A375 cells for 24h **(C)** and 48h **(D)**.

The cytotoxicity of free curcumin and Curcumin/MPEG-PLA micelles were investigated by MTT assay using B16 and A375 cells. As demonstrated in Figure 4, both free curcumin and Curcumin/MPEG-PLA micelles inhibited B16 and A375 proliferation in a time- and dose-dependent manner. With the drug concentration of Curcumin/MPEG-PLA micelles increasing from 0.16 μg/ml to 10 μg/ml, the relative cell viability of B16 decreased from 93.1 % to 48.6 % at 24 h (Figure 4A) and 93.2% to 26.1% (Figure 4B) at 48h. while With the drug concentration of Curcumin/MPEG-PLA micelles increasing from 0.16 μg/ml to 20 μg/ml, the relative cell viability of A375 decreased from 95.6% to 34.2% at 24h (Figure 4C) and 97.8 % to 25.5% (Figure 4D) at 48h. Meanwhile, the half maximal inhibitory concentration (IC_50_) of Curcumin/MPEG-PLA micelles (B16: 8.9 μg/ml; A375: 8.3 μg/ml) was lower than that of free curcumin (B16: 9.8 μg/ml; A375: 12.5 μg/ml) at 24h. The results revealed that Curcumin/MPEG-PLA micelles had cytotoxicity effect on melanoma cells and the encapsulation of curcumin in MPEG-PLA micelles improved its cytotoxic activity.

#### Apoptosis induced by curcumin or curcumin nanoparticles

**Figure 5:**
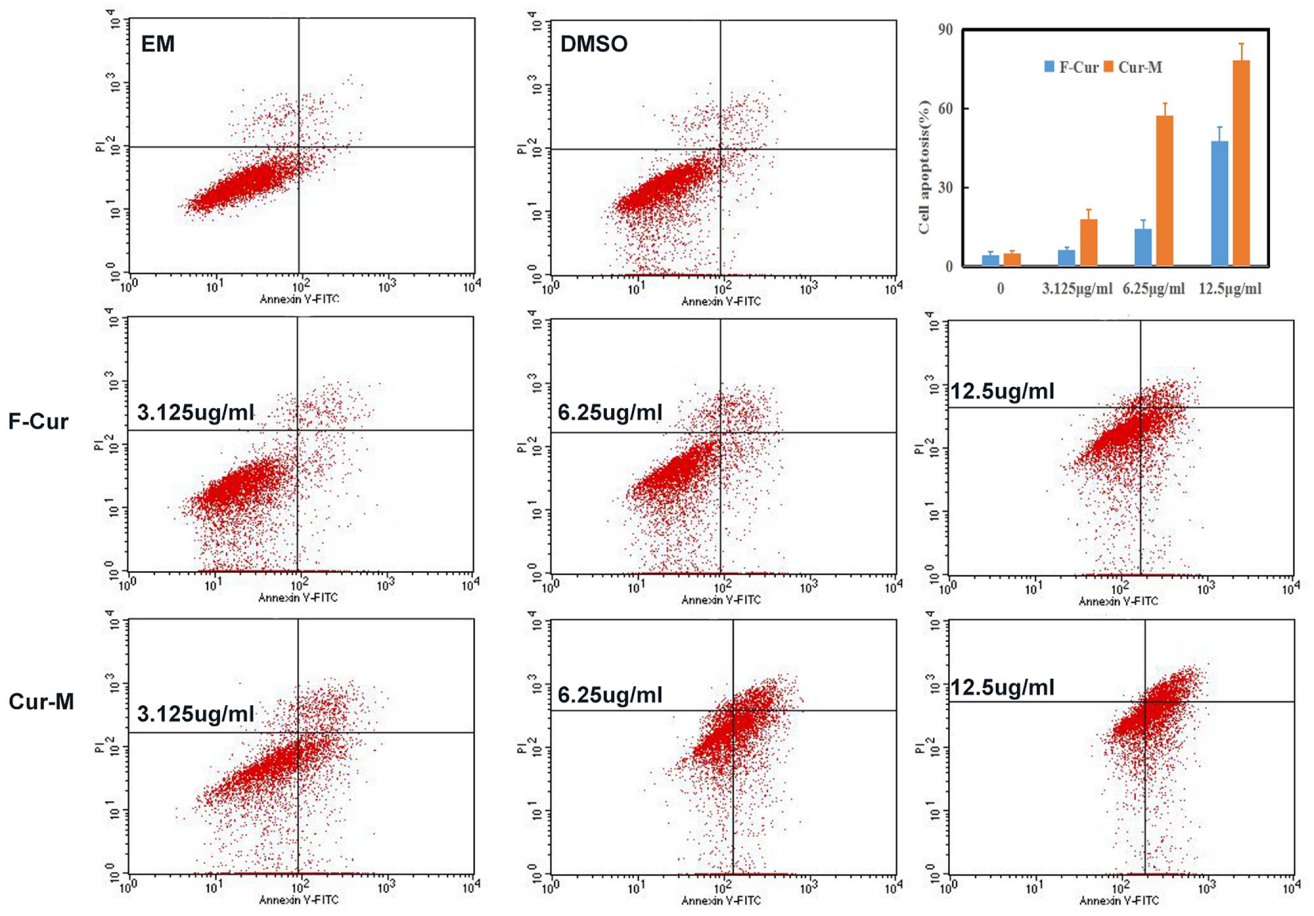
The determination of cells apoptosis by FCM 3.125μg/ml, 6.25μg/ml and 12.5μg/ml of free Curcumin and Curcumin/MPEG-PLA micelles were used to treat the melanoma cell B16 *in vitro*.

As apoptosis is an important mechanism of anti-cancer chemotherapy, we detected cell apoptosis of B16 cells induced by free curcumin and curcumin/MPEG-PLA micelles via annexin V-FITC and PI apoptosis assay. The ability of free curcumin and Curcumin/MPEG-PLA micelles to induce apoptosis of B16 cells was shown in Figure 5. In the NS and Blank micelle group, the apoptosis rate was 4.12% and 4.93%, respectively. In the free curcumin and Curcumin/MPEG-PLA micelles group, the apoptotic cells increased in a concentration-dependent manner. The apoptosis rates were 6.2% and 18.02%, 14.3% and 57.4%, 47.8 and 78.3% when the B16 cells were treated with free curcumin and Curcumin/MPEG-PLA micelles at a concentration of 3.12 μg/ml, 6.25 μg/ml and 12.5 μg/ml, respectively. It is obviously that Curcumin/MPEG-PLA micelle was capable of inducing more B16 cell apoptosis than other groups.

#### Enhanced curcumin uptake of melanoma cells from the curcumin nano micelles.

**Figure 6:**
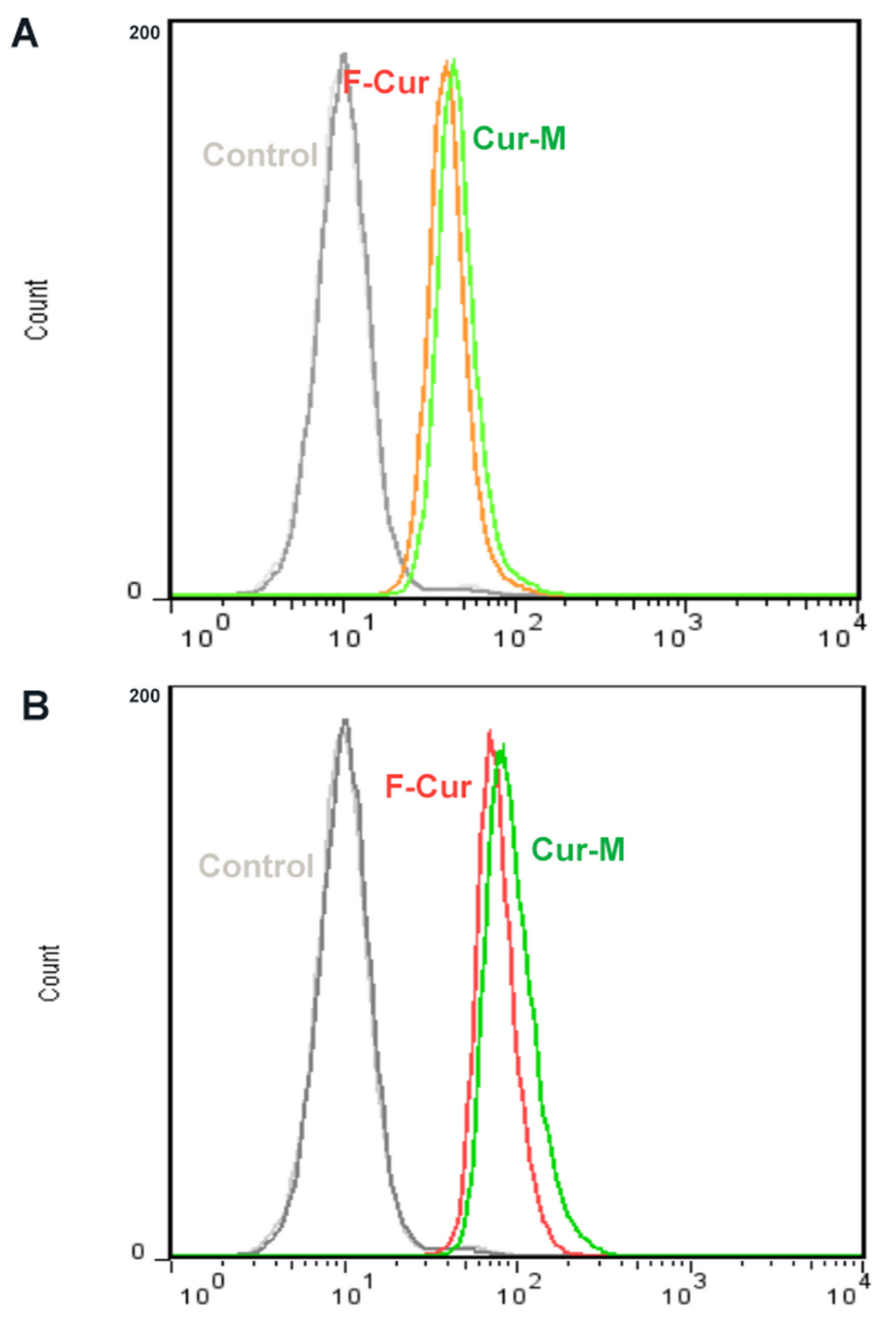
Cellular uptake of free Curcumin and Curcumin/MPEG-PLA Curcumin-derived fluorescence intensity of B-16 cells after 4 hours of exposure to 5 μg/ml **(A)** and 10 μg/ml **(B)** of curcumin or curcumin/MPEG-PLA micelles solution.

To investigate the mechanism of the improved cytotoxicity and apoptosis effect of Curcumin nano micelles, cellular uptake studies of Curcumin micelles or free Curcumin were performed on B16 cells. The results of the B16 cells treated with blank micelles as a control, free Curcumin, and Curcumin micelles at drug concentrations of 5 μg/ml and 10 μg/ml for 4 h are shown in Figure 6A and Figure 6B.

The enhanced cellular uptake of Curcumin micelles was also detected by FCM analysis. Figure 6 showed that the fluorescence intensity of cells treated with Curcumin micelles was much stronger than that in the free Curcumin group after incubation for 4h at different concentrations.

### Anti-melanoma effect *in vivo* of curcumin or curcumin nano-micelles

#### Curcumin and curcumin nanomicelles inhibited tumor growth in animal models

**Figure 7:**
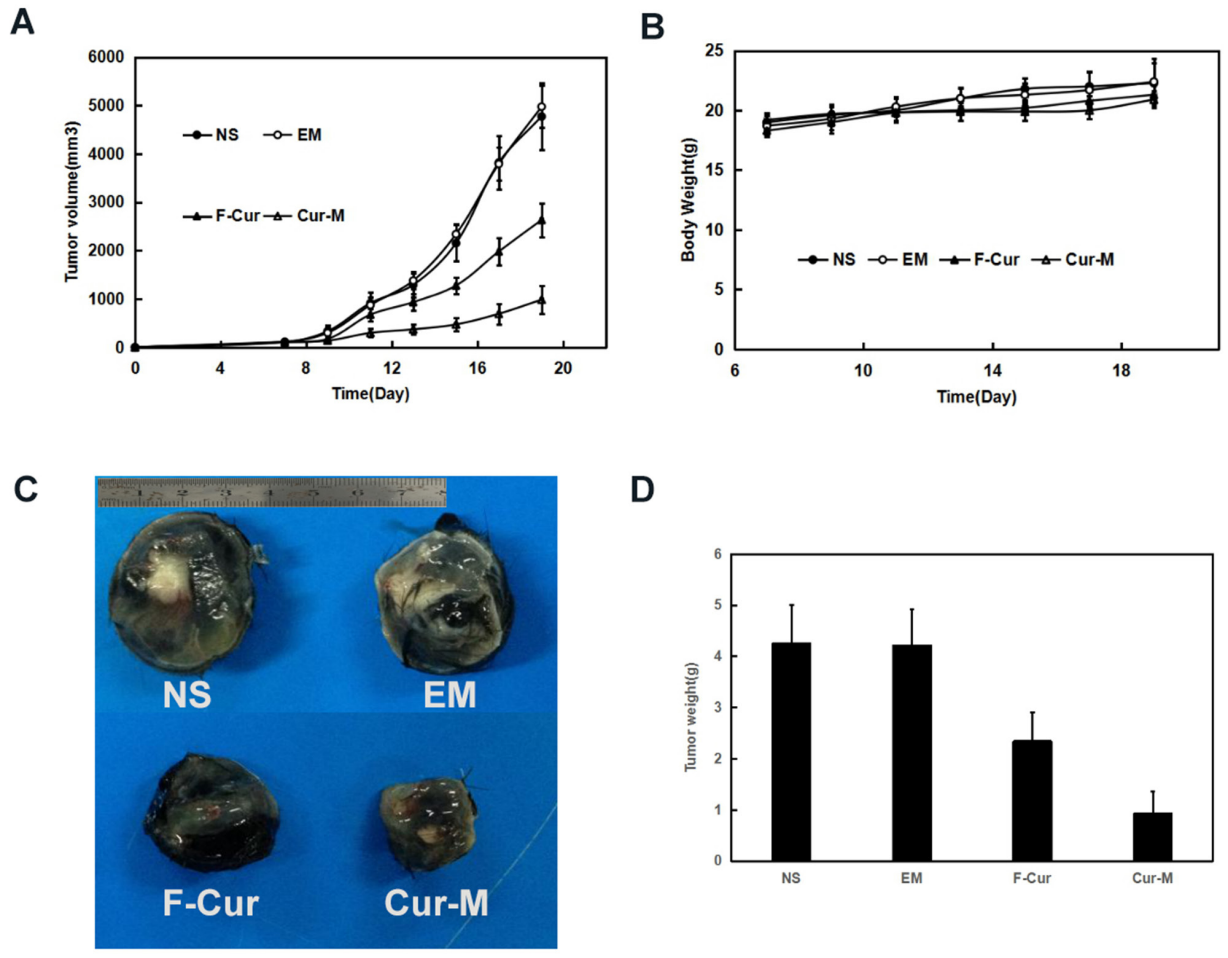
Anti-melanoma effect of Curcumin in subcutaneous B16 model In C57 mice xenograft model, C57 mice were injected subcutaneously with 0.1 mL of B16 cells containing 1×10^6^ cells in the right flank. As the tumor grows to about 0.1cm^3^, the mice were randomly assigned to 4 groups and treated with normal saline, blank micelles, free curcumin (Curcumin: 50mg/kg body weight) or Curcumin/MPEG-PLA (curcumin: 50mg/kg body weight) micelles everyday respectively. **(A)** Tumor volume curves. **(B)** Body weight. **(C)** Image of represent tumor in different groups. **(D)** The average tumor weight of each group.

In mice bearing B16 or A375 subcutaneous melanoma, curcumin (50mg/kg) exerted significant anti-melanoma effects without obvious impact on mice body weight. In mice bearing B16 subcutaneous melanoma model, the blank micelles had no anti-glioma effects. The tumor volumes decreased by 44.8 % and 79.4 % respectively under exposure of free curcumin and Curcumin/MPEG-PLA micelles in B16 subcutaneous melanoma model (p < 0.01, curcumin/MPEG-PLA micelles versus free curcumin) (Figure 7A). Free curcumin and curcumin/MPEG-PLA micelles exerted significant anti-melanoma effects without obvious impact on mice body weight (Figure 7B). The tumor weights in the curcumin/MPEG-PLA micelles group were lower than those of other groups in both animal models (Figure 7D). The tumor weights in the curcumin/MPEG-PLA micelles group were lower than those of other groups (p < 0.01, curcumin/MPEG-PLA micelles versus free curcumin) (Figure 7D).

**Figure 8:**
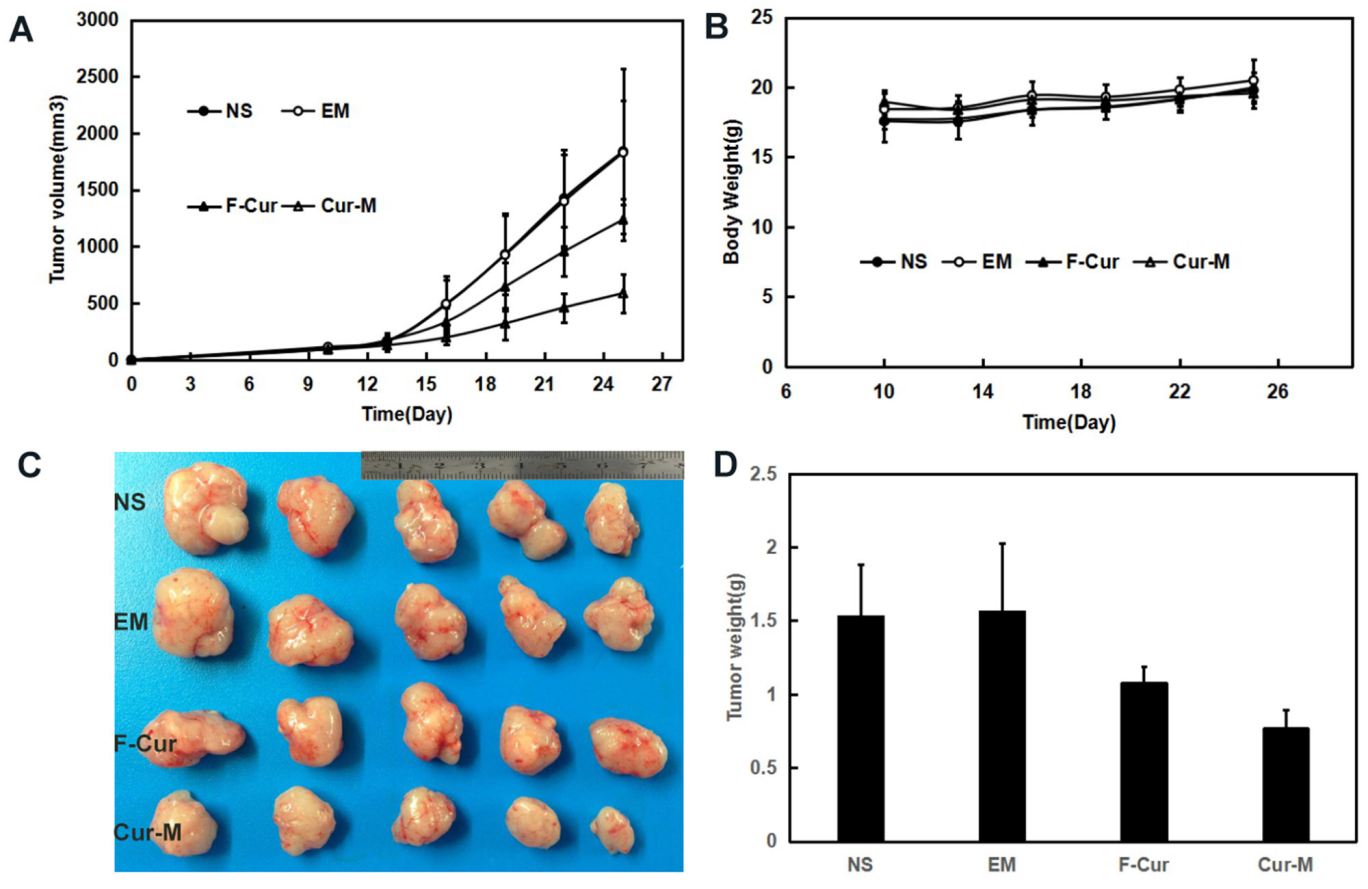
Anti-melanoma effect of Curcumin in subcutaneous A375 model In the mice xenograft models, mice were injected subcutaneously with 0.1 mL of A375 cells containing 1×10^7^ cells in the right flank. As the tumor grows to about 0.1cm^3^, the mice were randomly assigned to 4 groups and treated with normal saline, blank micelles, free curcumin (Curcumin: 50mg/kg body weight) or Curcumin/MPEG-PLA (curcumin: 50mg/kg body weight) micelles everyday respectively. **(A)** Tumor volume curves. **(B)** Body weight. **(C)** Tumor photo of each group. **(D)** The average tumor weight of each group.

In mice bearing A375 subcutaneous melanoma model, the tumor volumes decreased by 32.8 % and 68.4 % respectively under exposure of free curcumin (F-cur) (50mg/kg) and curcumin/MPEG-PLA micelles (Cur-M) (50mg/kg) in A375 subcutaneous melanoma with the same interventions (p<0.01, curcumin/MPEG-PLA micelles versus free curcumin) (Figure 8A). Free curcumin and curcumin/MPEG-PLA micelles exerted significant anti-melanoma effects without obvious impact on mice body weight (Figure 8B). The tumor weights in the curcumin/MPEG-PLA micelles group were obviously lower than those in other groups in both animal models (Figure 8D). The tumor weights in the curcumin/MPEG-PLA micelles group were lower than those of other groups (p < 0.01, curcumin/MPEG-PLA micelles versus free curcumin) (Figure 8D).

#### Histological analysis by TUNEL, Ki67 and CD31assays

**Figure 9:**
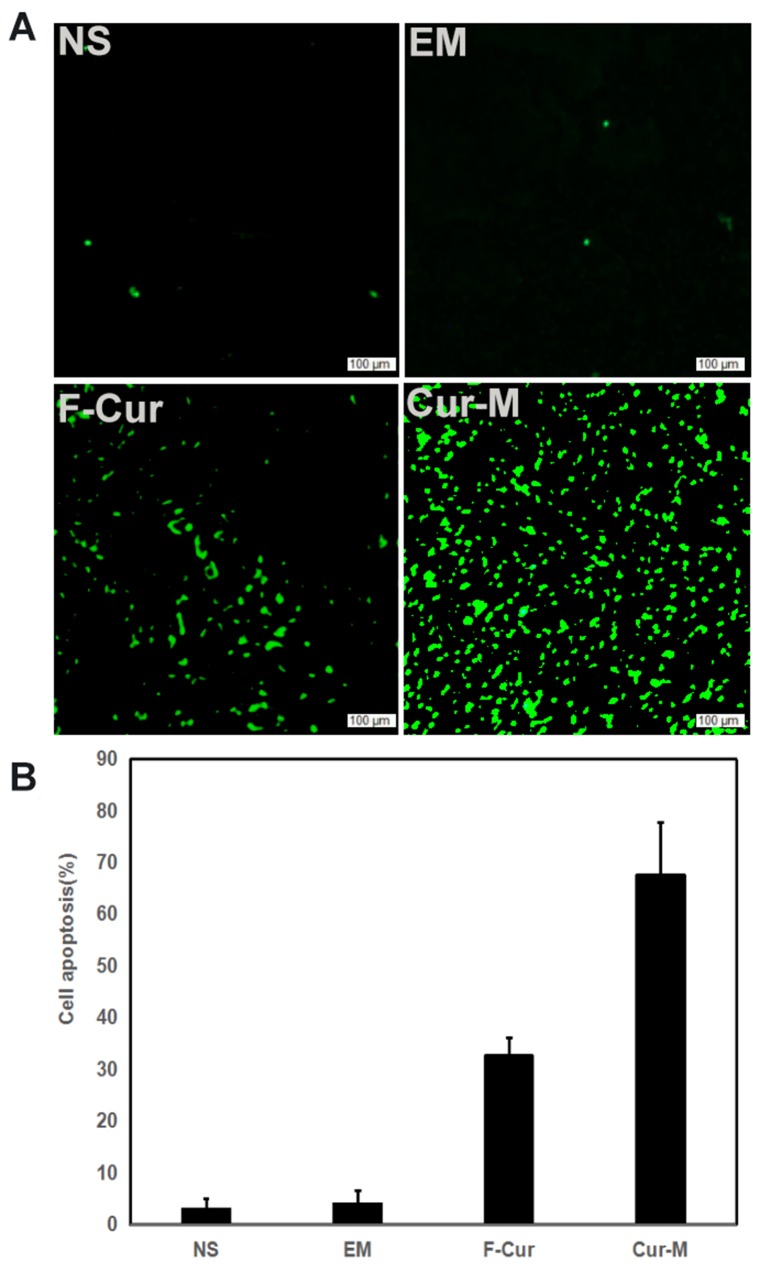
Cell apoptosis examined using TUNEL analysis When A375 tumor tissues were sectioned, a TUNEL kit was used to analyze apoptotic cells within tumors. **(A)** Representative images of each group. **(B)** Mean apoptosis cells every five fields.

Tumor sections were stained with TUNEL to investigate cell apoptosis in four groups, which were treated with NS, blank micelles, free curcumin and Curcumin/MPEG-PLA micelles, respectively. As shown in Figure 9, no considerable positive nuclei was found in the NS and blank micelles group while substantial amount of TUNEL stains were observed in the curcumin/MPEG-PLA micelles group. The apoptotic index was 3% ± 2% in the NS group, 4 % ± 2.6 % in the blank micelle group, 32.7 % ± 3.5 % in the free curcumin group (P < 0.05) and 67 ± 10 % in the Curcumin/MPEG-PLA micelles group (P < 0.05). The results indicated that curcumin encapsulated in the MPEG-PLA micelles was more capable of inducing apoptosis of A375 cells *in vivo*.

**Figure 10:**
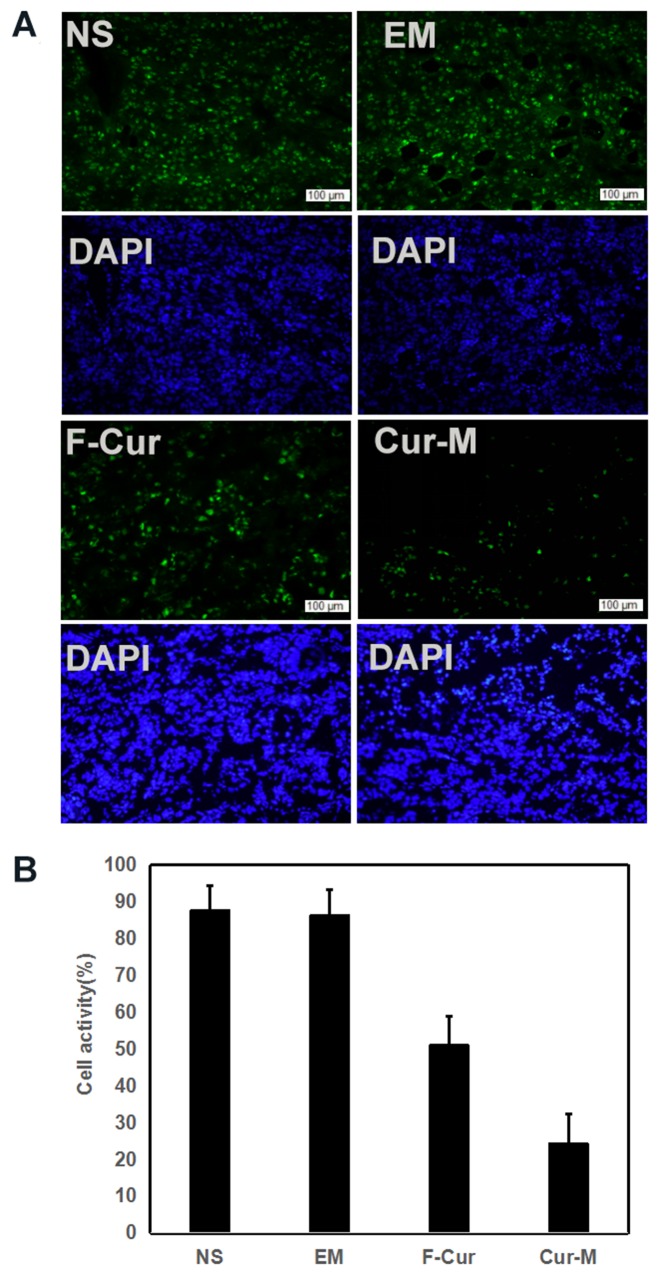
Cell proliferation activity detection When A375 tumor tissues were sectioned, Ki67 antibody was used to analyze cells proliferation activity within tumors. **(A)** Representative images of Ki-67 staining. **(B)** Mean Ki-67 positive cells every five fields.

The A375 tumor cell proliferation was evaluated by immunohistochemical staining. Tumor sections from four groups were stained with Ki-67. As shown in Figure 10, the positive cells which emitted red fluorescence were abundant in the NS group, blank micelle group and free curcumin group. In comparison, few positive cells were observed in the Curcumin/MPEG-PLA micelles group. The Ki-67 labelling index, which is usually related to the clinical course of tumor, is significantly lower in Curcumin/MPEG-PLA micelles group (24.3 % ± 8.1 %) than other groups (87.7 % ± 6.8 % in NS group, P < 0.05; 86.3% ± 7.1 % in blank micelle group, P < 0.05; 51 % ± 8 % in free Curcumin group, P < 0.05). It is evident that Curcumin/MPEG-PLA micelles effectively suppressed tumor proliferation.

**Figure 11:**
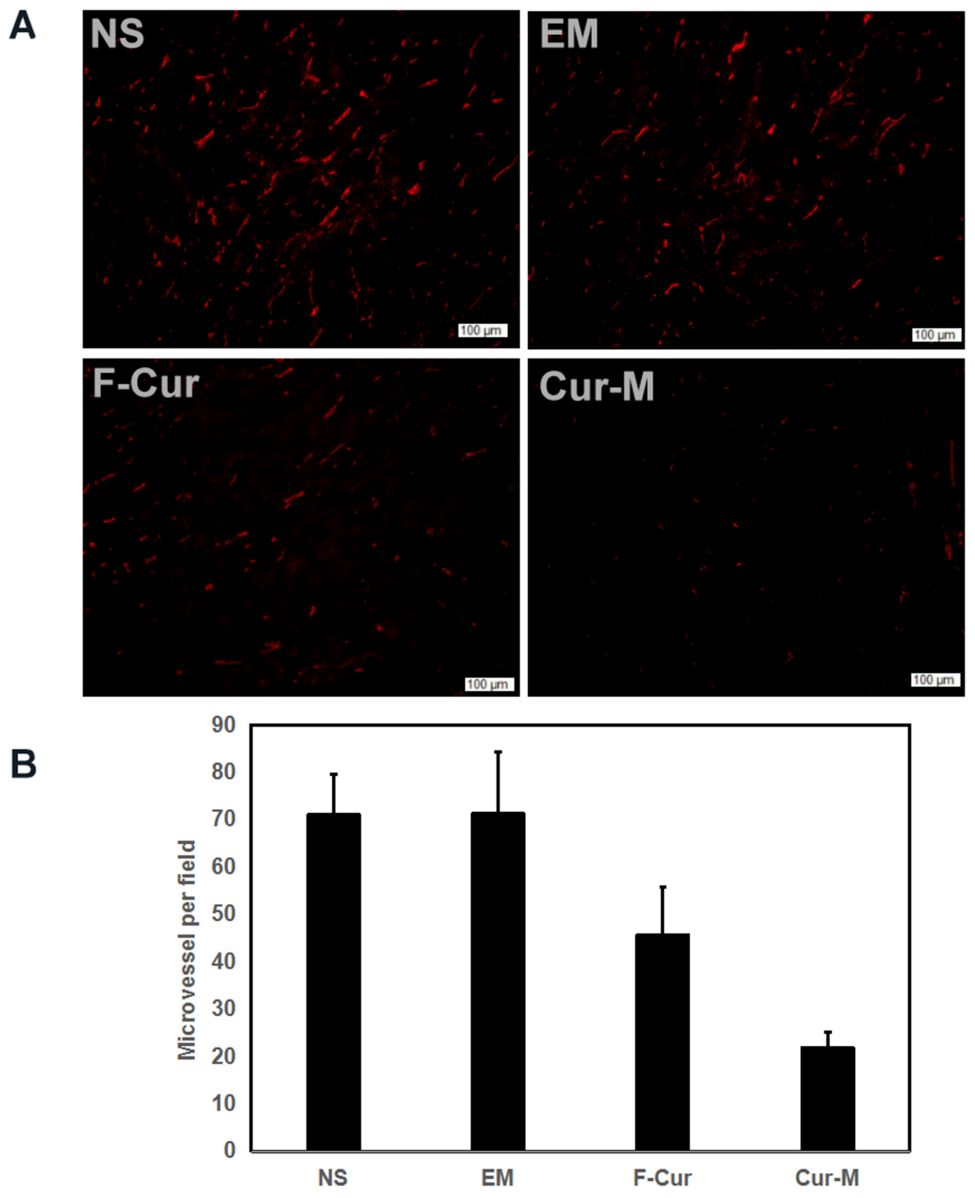
CD31 immunofluorescent staining When A375 tumor tissues were sectioned, CD31 antibody was used to analyze cells proliferation activity within tumors. **(A)** Representative CD31 immunofluorescent staining of each group. **(B)** MVD every five fields.

Furthermore, the angiogenesis of A375 melanoma was explored. As shown in Figure 11, few microvessels with red fluorescence were observed in the group treated with Curcumin/MPEG-PLA micelles. The number of microvessels was significantly lower in the Curcumin/MPEG-PLA micelles group (21.2 ± 3.4) compared with NS group (71 ± 8.5, P<0.05), blank micelles group (71 ± 13, P<0.05) and free curcumin group (45.6 ± 10, P<0.05). We propose that the mechanism of anti-angiogenesis may play an important role in inhibiting tumor growth by Curcumin/MPEG-PLA micelles.

### Curcumin and curcumin nanoparticles inhibit tumor angiogenesis

**Figure 12:**
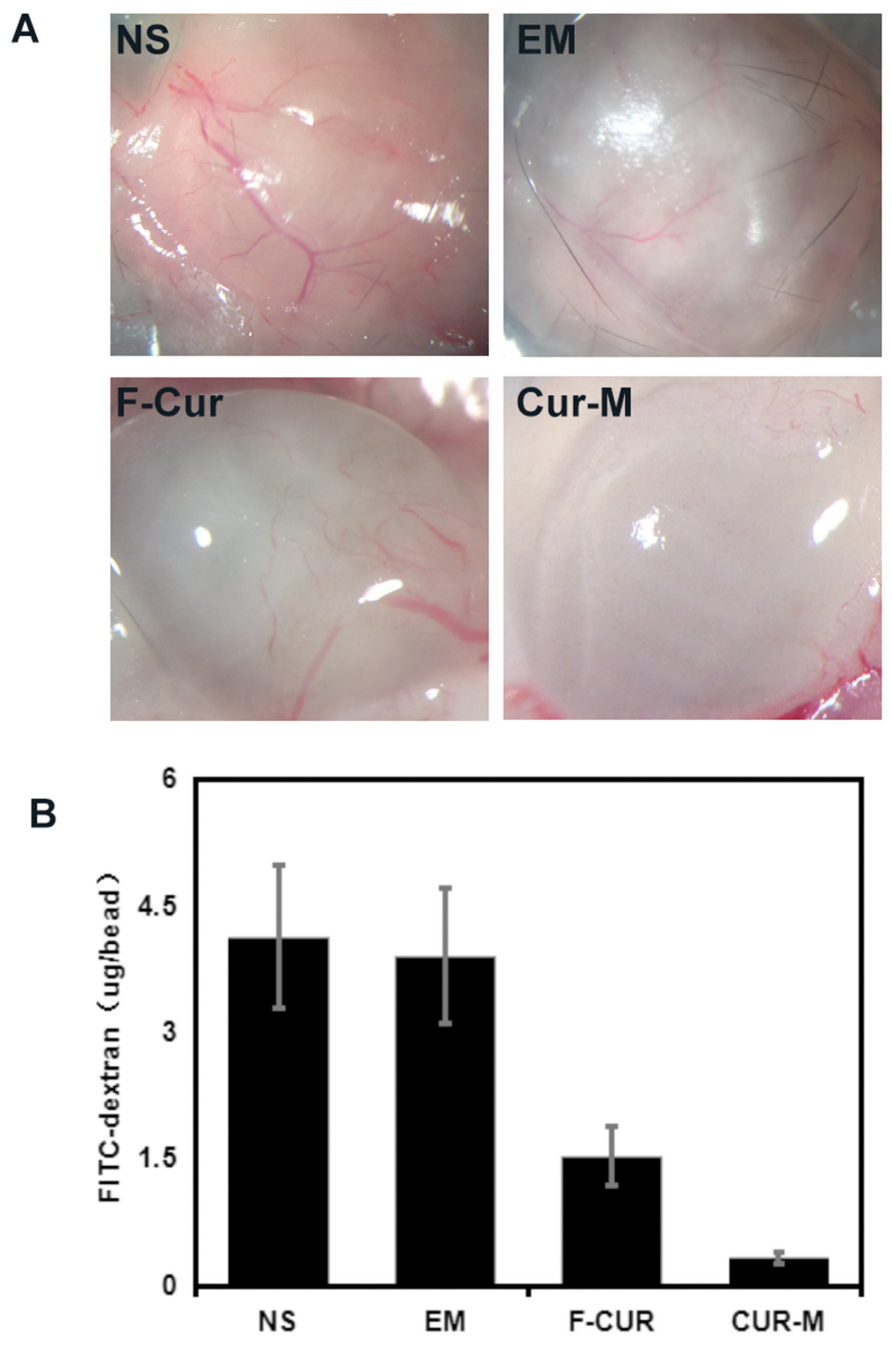
Vasculation of alginate implants Alginate beads containing B16 cells were implanted subcutaneously into the mice. Beads were removed after treatment by free curcumin and Curcumin/MPEG-PLA micelles. **(A)** Images of each group. **(B)** uptake of FITC–dextran in each group.

The inhibitions of angiogenesis by curcumin and curcumin nanoparticles were determined by an alginate-encapsulated B16 tumor cell assay. As shown in Figure 12, alginate implant angiogenesis can be directly observed. The vascularization of the bead with curcumin nanoparticles treatment was clearly suppressed compared with that of other groups. The alginate implant angiogenesis was also quantified by measuring the uptake of FITC–dextran into beads.

The FITC–dextran uptake was significantly lower in the mice treated with curcumin nanoparticles (0.33±0.06 μg per implant) compared with free curcumin (1.53±0.35 μg per implant, P <0.01), blank micelles (3.93±0.81 μg per implant, P < 0.01), and NS (4.1±0.83 μg per implant, P < 0.01) groups.

## DISCUSSION

Curcumin is a kind of pigment extracted from turmeric plant, and it also exists in other ginger plants. Curcumin has good anti-inflammatory activity and is used in the treatment of various diseases such as wound-healing, ulcers, and arthritis [15–17].

Many studies have reported that curcumin could prevent tumorigenesis and inhibit growth of implanted human tumors in the mouse model [41, 42]. Therefore, curcumin would be an ideal anti-melanoma agent if it can efficaciously block tumor growth or directly kill glioma cells. Unfortunately, many studies have revealed that one of the major problems with curcumin is its low bioavailability *in vivo*. Traditionally, turmeric is delivered orally as an emulsion in oil or milk; perhaps because of the hydrophobic nature of its bioactive constituents such as curcumin and turmeric oil.

Oral administration of curcumin is not reasonable due to its low bioavailability caused by the poor absorption and first pass effect. Recent research showed that the oral absorption rate of flavonoids is less than 10%[43, 44]. Moreover, curcumin is prone to be eliminated in circulation. Therefore, it is hard to achieve effective plasma concentration of curcumin applied either orally or by injection.

Effective chemotherapy requires a continuously high local concentration of drugs in the tumor site. The diameter of curcumin/MPEG-PLA micelles measured by DLS in our research was 34.5 nm. Since the interendothelial junctions of tumor mainly vary from 40 nm to 80 nm, curcumin/MPEG-PLA micelles were able to enter the tumor region through passive diffusion with little hindrance. Together with the impaired lymph drainage of nano-carriers in tumor, the enhanced penetration and retention (EPR) effect was greatly increased.

In this work, the self-assembly characteristics of MPEG-PLA diblock copolymers were used to prepare curcumin nanoparticles [39]. The drug loading of curcumin in nanoparticle reached 10% and the packing rate reached 98.5%, which was consistent with previously reports [39]. In this study, the zeta of Curcumin/MPEG-PLA nanoparticles was slightly negative. As the surface of plasma and blood cells has negative charge, negative charge and negative charge are mutually exclusive under role of electric field. Thus the surface of curcumin drug-loaded nanoparticles with a slight negative charge is not easy to occur adhesion, adsorption and cluster with the blood cells and Plasma. Nano-particles also maintain mutually exclusive state in the electric field, extend circulation time in the blood to improve efficacy.

A large number of studies have shown that curcumin has a broad-spectrum anti-tumor effect [18–27]. Curcumin can not only directly inhibit tumor cell proliferation but also induce cell apoptosis by regulating cell cycle, activating endogenous and exogenous pathways. What is more, curcumin have anti-tumor neovascularization and chemotherapy sensitization effect and other effects. The anti-tumor effect of curcumin has many characteristics such as multi-target, multi-link and multi-channel, which involves in the complicated signal transduction pathway and regulation mechanism. In this research, we were focused on the anti-tumor effect of curcumin, inducing tumor cell apoptosis and anti-tumor neovascularization. The results of MTT assay indicated that both free curcumin and curcumin nanoparticles could inhibit melanoma growth which was time and drug concentration dependent. The IC_50_ of curcumin nano-particles on the B16 and A375 cells was 8.9 μg/ml and 8.3 μg/ml, respectively. From cell apopotosis test, we found that the curcumin nanoparticle could induce more cell apoptosis than free curcumin. This may be caused by the high water soluble and good biocompatibility. Through a number of anti-melanoma experiments *in vivo*, we found that curcumin has many anti-tumor effects in the treatment of melanoma.

In summary, MPEG-PLA nanoparticles have good biosafety, which is an ideal carrier to deliver curcumin. The water-soluble and pharmacokinetic characteristics of curcumin have been improved by nano-particles after MPEG-PLA packaging. And the anti-melanoma effect of curcumin was increased by nano-particle. The curcumin/MPEG-PLA micelles have the potential clinical application in melanoma chemotherapy.

## MATERIALS AND METHODS

### Reagents

Curcumin, poly (ethylene glycol) (PEG, Mn = 4000), anhydrous L-lactide, stannous octoate (Sn(Oct)_2_), 3-(4, 5-dimethylthiazol-2-yl)-2, 5-diphenyl tetrazolium bromide (MTT) were purchased from Sigma (St Louis, MO, USA). Annexin V-FITC/PI Detection kit was purchased from keyGEN Biotech, China. Both Dulbecco’s Modified Eagle’s Medium (DMEM) and fetal bovine serum (FBS) were purchased from Gibco (USA). All other chemicals were purchased from Kelong Chemicals (Chengdu, China). All the chemicals used in this work were analytical reagent (AR) grade, and used without further purification except PEG.

Female C57BL/6 mice and female BALB/c nude mice (18 ± 2g) were purchased from the Laboratory Animal Center of Sichuan University (Chengdu, China). All studies involving mice were performed following the protocol approved by the Institutional Animal Care and Treatment Committee of Sichuan University (Chengdu, P.R. China). All the animals were treated humanely during the experiment.

### Cell lines

The murine melanoma cell line, B16F10 and human melanoma cell line A375 were purchased from American Type Culture Collection (ATCC, Manassas, VA), and grew in DMEM medium supplement with 10% FBS at a 37°C incubator with a humidified 5% CO_2_ atomosphere. Culture media were supplemented with 100 U/mL penicillin and 100 mg/mL streptomycin.

### Formulation of nanoparticles loading curcumin

Curcumin nanoparticles were prepared by one-step self-assembly method. First, 10mg curcumin and 90mg MPEG-PLA were dissolved in acetone solution. Second, the mixture was added in the normal salt solution under constant stirring for 5 minutes. At last, the resulting dispersion of nanoparticles was vacuum evaporated to eliminate the organic solvent. The resultant formulation was called Curcumin/MPEG-PLA micelles solution. Finally, the prepared Curcumin/MPEG-PLA micelles were lyophilized and stored at 4 °C.

### Drug loading (DL) and encapsulation efficiency (EE)

We dissolved lyophilized Curcumin/MPEG-PLA micelles (10 mg) in methanol (0.1 ml) to determine the concentration of curcumin. The content of curcumin in the formulation was detected by high performance liquid chromatography (HPLC, Shimadzu LC-20AD, Japan). Drug loading (DL) and encapsulation efficiency (EE) of Curcumin/MPEG-PLA micelles were calculated by the following formulas (1) and (2):

$$\mathrm{DL=}\frac{\mathrm{Curcumin}}{\mathrm{Polymer}+\mathrm{Curcumin}}\text{×}100\text{\%}$$ (1)

$$\mathrm{EE=}\frac{\mathrm{Experimental}\;\mathrm{Curcumin loading}}{\mathrm{Theoretical}\;\mathrm{Curcumin}\;\mathrm{loading}}\text{×}100\text{\%}$$ (2)

### Dynamic light scattering (DLS) measurements

Dynamic light scattering (DLS, Malvern Nano-ZS 90) was used to measure the particle size and zeta potential of Curcumin/MPEG-PLA micelles at 25 °C after previous dilution of samples with distilled water. This process was performed in triplicate.

### Transmission electron microscopy (TEM)

Transmission electron microscopy (TEM, H-6009IV, Hitachi, Japan) was applied to determine the morphological characteristics of Curcumin/MPEG-PLA micelles. In brief, diluted suspension of Curcumin/MPEG-PLA micelles was Placed drop-wise on a copper TEM grid. Then the grid was negatively stained with phosphotungsten acid (2%, w/v) for 20 minutes and allowed to dry. Thirty particles were observed to calculate the mean particle diameter.

### Release *in vitro*

The dialysis bag method was employed to evaluate the *in vitro* release kinetics of curcumin from Curcumin/MPEG-PLA micelles. Briefly, the dialysis bags (Sigma, St. Louis, MO, USA) which contained 1ml of Curcumin/MPEG-PLA micelles were incubated in 200 ml phosphate buffer solution (PBS, pH 7.4) with 0.5% Tween-80 at 37 °C under gentle stirring. At different time points, 200 ul of the external medium was replaced with fresh medium. Then the curcumin content in samples were quantified by HPLC system. This process was repeated in triplicate.

### Pharmacokinetics

Pharmacokinetics investigation was conducted in Male Sprague-Dawley rats. The rats were randomly divided into two groups of five animals each. The two groups were injected with free curcumin (curcumin: 50mg/kg) or curcumin/MPEG-PLA micelles (curcumin: 50mg/kg) respectively through the tail vein injection. At predetermined time points (predose, 15min, 30min, 1h, 2h, 4h, 8h, 12h, 24h), the blood was collected from carotid artery and serum concentration of curcumin was analyzed by HPLC.

### MTT test

The effects of free curcumin and curcumin/MPEG-PLA micelles on B16 and A375 cell viability were evaluated by MTT method. In brief, B16 and A375 cells were seeded at a density of 5×10^3^ cells per well in 96-well plates and allowed to grow in DMEM added with 10% FBS for 24h. Cells were then treated with free curcumin or curcumin/MPEG-PLA micelles at different concentrations respectively for 24h or 48h. At the end of incubation, fresh medium containing 500 μg/ml MTT was added. Thereafter cells were incubated for 3h at 37 °C. The formazan was then dissolved in DMSO during the process and the absorbance was measured at 570 nm using a plate reader (OPTImax, Molecular Dynamics). This process was repeated in triplicate.

### Apoptosis study

Induction of apoptosis of A375 cells by free curcumin or curcumin/MPEG-PLA micelles was investigated by flow cytometry (FCM, BD FACSCalibur). In brief, A375 cells were seeded at at density of 5×10^5^ cells per well in 6-well plates. The next day, cells were treated with free curcumin or curcumin/MPEG-PLA micelles at different concentrations for 24h. After incubation cells were harvested and stained with 5μl Annexin-V-FITC and 5μl PI (Annexin V-FITC/PI Apoptosis Detection Kit). Then the apoptosis of stained cells were analyzed in a flow cytometry. This process was repeated in triplicate.

### Cellular uptake assay

B16 cells maintained in DMEM supplemented with 10% fetal bovine serum and 100 U mL^-1^ penicillin/streptomycin were cultured at 37 °C and 5% CO_2_. The cells were seeded in six well plates (MatTek, USA) at a seeding density of 2.5 x 10^5^ cells per well in 1 mL of growth medium. The cells were incubated for 24 h, and then the growth medium was replaced with the fresh one containing either free curcumin or drug-encapsulated micelles. The curcumin content was set at 5 and 10 μg/mL and pure drug dissolved in dimethylsulfoxide was used as a control. After incubation for 4 h, the cells were washed with PBS three times. Taking advantage of the intrinsic green fluorescence of curcumin, flow cytometry was employed to analyse uptake of curcumin in the B16 cells.

### Anti-melanoma effect *in vivo*

The anti-melanoma effects Curcumin/MPEG-PLA micelles were studied in C57 mouse and nude mice xenograft model. In C57 mice xenograft model, C57 mice were injected subcutaneously with 0.1 mL of B16 cells containing 1×10^6^ cells in the right flank. In the mice xenograft models, mice were injected subcutaneously with 0.1 mL of A375 cells containing 1×10^7^ cells in the right flank. As the tumor grows to 0.1cm^3^, the mice were randomly assigned to 4 groups and treated with normal saline, blank micelles, free curcumin (Curcumin: 50mg/kg body weight) or Curcumin/MPEG-PLA (Curcumin: 50mg/kg body weight) micelles everyday respectively. The diameter of tumor was assessed by caliper in two dimensions every other day and the body-weight of mice was measured in the meanwhile. Tumor volume was calculated according to the following formula: V=0.52^*^(ab^2^), where a represented the length while b represented the width. Mice were sacrificed at predetermined time and tumors were immediately weighed.

### TUNEL assay

Apoptosis of A375 tumors induced by Curcumin/MPEG-PLA micelles was detected by TUNEL staining. When tumor tissues were sectioned, a TUNEL kit (Promega, Madison, WI, USA) was used to analyze apoptotic cells within tumors following the manufacturer’s protocol. Five equal-sized tumor sections were detected. TUNEL positive cells were observed under a fluorescent microscope (×400).

### Immunohistochemical examination of Ki67 and CD31

Tumor proliferation activity and neovascularization in tumor tissues were investigated by Immunohistochemical analysis with antibody Ki67 and CD31. Tumor sections were stained with rabbit anti-mouse ki67 polyclonal antibody (1: 50; BD PharmingenTM, USA) or rabbit anti-mouse CD31. Then they were washed twice with PBS, and incubated with FITC- or Rhodamine-conjugated secondary antibody respectively for 1h (Abcam, USA). Ki-67 positive and total cells were counted in each tumor section under microscope and Ki-67 labeling index was calculated. The CD31 positive microvessels were counted under high power field (×400).

### Alginate-encapsulated tumor induced vascular experiment

An alginate-encapsulated tumor cell assay was performed as described previously [39]. B16 cells were resuspended in a solution of alginate (1.5% wt) and dropped into a solution of calcium chloride (250 mM), forming alginate beads containing 1×10^5^ tumor cells per bead. The bead was then implanted subcutaneously into an incision made on the dorsal side of the mouse on day 0. Twelve bead-bearing mice were divided into four groups (3 mice per group), and treated with Curcumin/MPEG-PLA (Curcumin: 50 mg/kg), free curcumin (Curcumin: 50 mg/kg), blank micelles (control) or normal salt (NS) for 5 days, respectively. On day 12, mice were injected intravenously with 0.1 mL of a 100 mg/kg FITC-dextran solution. Alginate beads were photographed and rapidly removed 20 min after FITC-dextran injection. The uptake of FITC-dextran was measured as described.

### Statistical analysis

Results were expressed as mean ± SD. Statistical analysis was performed with one-way analysis of variance (ANOVA) using SPSS 15.0 software (Chicago, IL, USA). Values of P < 0.05 are indicative of statistically significant.
